# Transient and unique lesion of acantholytic dyskeratosis of the axilla

**DOI:** 10.1016/j.jdcr.2024.12.007

**Published:** 2024-12-16

**Authors:** Valérie Bédard, Justin Deschamps, Jessika Hétu, Dominique Hanna

**Affiliations:** a Department of Surgery, University of Sherbrooke, Centre intégré universitaire de santé et de services sociaux (CIUSSS) de l’Estrie-Centre hospitalier universitaire de Sherbrooke (CHUS), Sherbrooke, Quebec, Canada; b Department of Pathology, University of Sherbrooke, Centre intégré universitaire de santé et de services sociaux (CIUSSS) de l’Estrie-Centre hospitalier universitaire de Sherbrooke (CHUS), Sherbrooke, Quebec, Canada; c Division of Dermatology, Department of Medicine, University of Sherbrooke, Centre intégré universitaire de santé et de services sociaux (CIUSSS) de l’Estrie-Centre hospitalier universitaire de Sherbrooke (CHUS), Sherbrooke, Quebec, Canada

**Keywords:** acantholysis, Darier, dyskeratosis, Grover, Hailey-Hailey, transient acantholytic dyskeratosis

## Introduction

Acantholytic dyskeratosis has a distinctive histologic pattern characterized by the following localized pathologic changes in the epidermis: (1) acantholysis with suprabasilar clefts, (2) acantholytic and dyskeratotic cells, and (3) hyperkeratosis and/or parakeratosis.^1^ Transient acantholytic dyskeratosis (Grover disease) is usually characterized clinically by a pruritic papulovesicular eruption. The precise origin has not yet been defined.^2^ A unique plaque is not a usual clinical presentation.

Acantholytic dyskeratosis is an uncommon diagnostic entity apart from the main acantholytic disorders, such as Grover disease (transient acantholytic dyskeratosis),^2^ Darier disease (keratosis follicularis),^3^ and Hailey-Hailey disease (benign familial pemphigus).^4^ A recent form attributed to COVID-19 vaccine^5^ has been described, and it can also be an incidental finding in a wide variety of conditions.

## Case presentation

**Fig 1:**
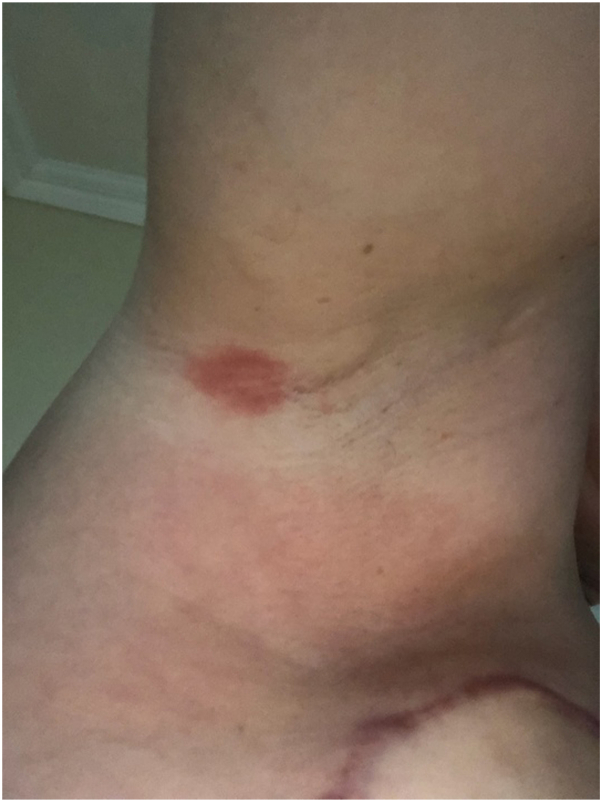
Right axillary erythematous plaque, week 2.

A 49 year-old woman presented to the dermatology clinic in March 2024 with a new red isolated 2-cm erythematous and pruriginous plaque of the right axilla (Fig 1).

The lesion progressed over 3 weeks despite application of polymyxin B-gramicidin (Polysporin) antibiotic ointment. The patient had a history of ipsilateral breast cancer (T3N0, stage III, in June 2022) treated with neo-adjuvant chemotherapy (Destiny-11 Arm B: trastuzumab deruxtecan followed by paclitaxel/trastuzumab/pertuzumab), total mastectomy, adjuvant chemotherapy (trastuzumab), and radiation therapy. The radiation therapy field was limited to the breast area, not involving the axilla at all. The patient also had a deep inferior epigastric perforator flap reconstruction in September 2023 before the lesion appeared. No history of skin, nail, or hair disease was reported. Only a change in her exercise training program was noted. The review of systems was negative and the complete skin examination did not show any other lesion. There was no family history of skin diseases.

**Fig 2:**
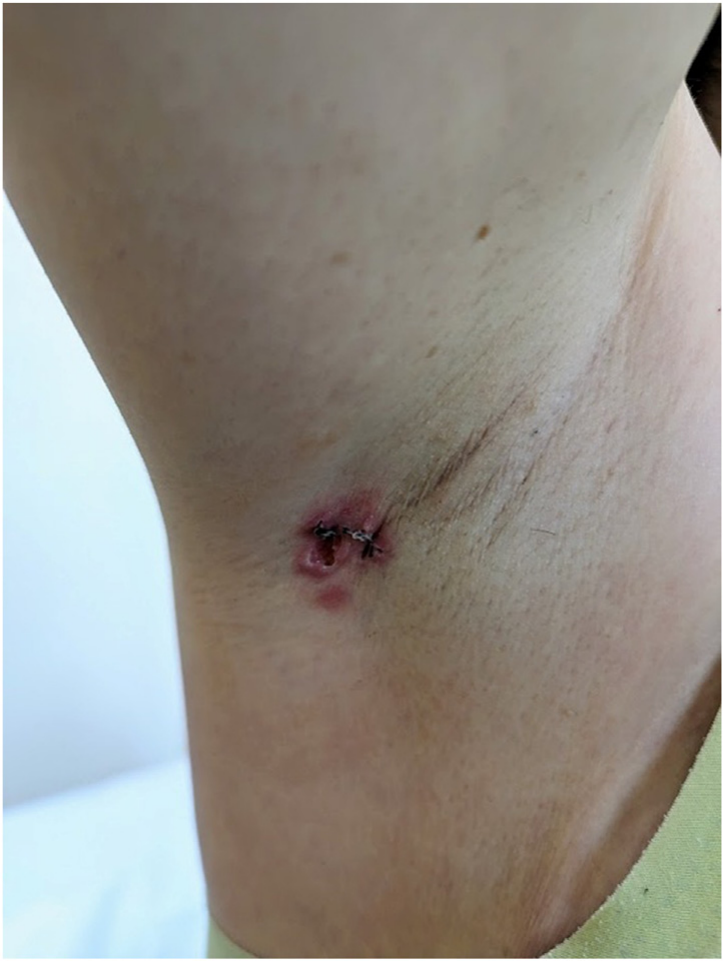
Right axillary erythematous plaque, week 3 (1 week postbiopsy).

Two punch biopsies were performed after 2 weeks of progression(Fig 2) to rule out loco-regional breast cancer recurrence.

**Fig 3:**
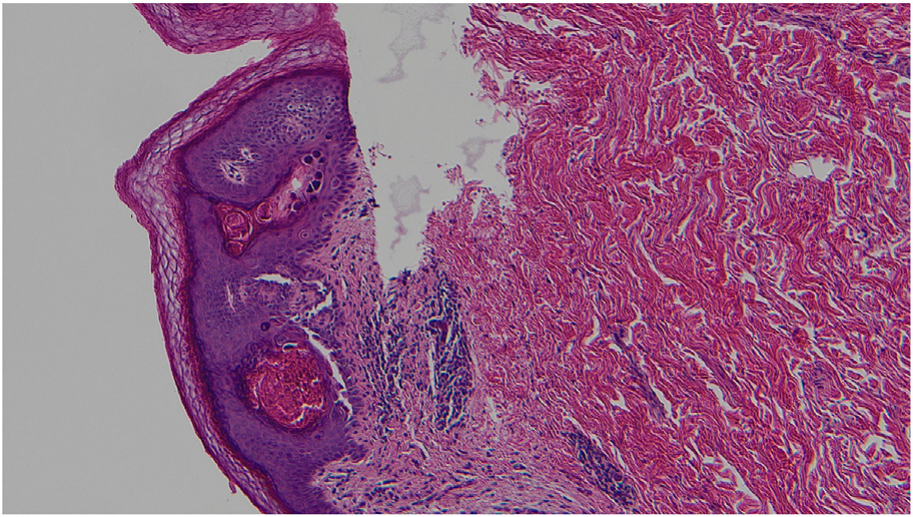
Hematoxylin and eosin-stained slide showing acantholytic dyskeratosis with suprabasilar clefts with corps ronds and corps grains. (Hematoxylin-eosin stain; original magnification: ×100.)

**Fig 4:**
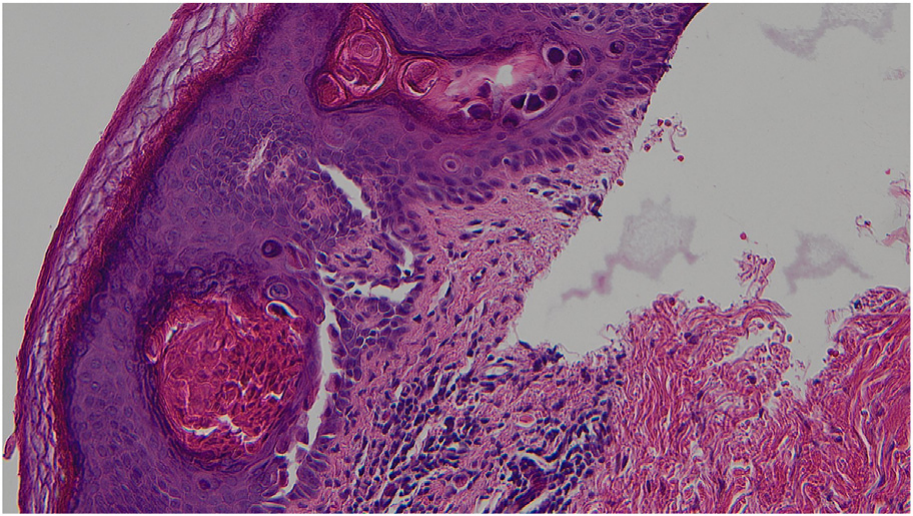
A higher magnification view of Figure 3. (Hematoxylin-eosin stain; original magnification: ×200.)

The initial histopathologic examination was done by a breast pathologist who only described a benign epidermoid cyst. The patient was referred to the dermatology clinic for management of the residual lesion. As the clinical presentation did not correlate with the diagnosis of an epidermal cyst, the slides were reviewed by a dermatopathologist with a corrected diagnostic of acantholytic dyskeratosis. With deeper levels cut in the biopsies’ cell blocks, the histopathologic examination demonstrated suprabasilar clefts in the epidermis with discohesive rounded keratinocytes (acantholysis). There was also dyskeratosis with 2 types of dyskeratotic cells, one type consisting of hypereosinophilic keratinocytes with pyknotic nuclei (*corps grains*) and the other type consisting of acantholytic keratinocytes with a larger nucleus and a perinuclear halo (*corps ronds*) (Figs 3 and 4). The skin appendages were spared by the acantholytic dyskeratosis.

Since the lesion was oozing, the treatment was changed to fusidic acid antibiotic cream and 1% hydrocortisone cream twice a day, with advice to keep the area dry and to remove any potential exacerbating factor (sweating, friction, and maceration). The itchiness resolved over the following days and the erythema improved over the following weeks. The lesion completely resolved after 1 month.

## Discussion

Acantholysis is a histologic phenomenon defined by the loss of intercellular cohesion between keratinocytes, resulting in cell separation and a rounded cellular outline. When associated with apoptosis and premature keratinization of individual keratinocytes, the term “acantholytic dyskeratosis” is used instead. It may or may not be accompanied by clinically apparent blisters. It is important to consider in the differential diagnosis diseases that are frequent mimickers,^6^ for example, infectious or inflammatory conditions, benign keratoses with acantholysis (such as actinic keratosis or acantholytic acanthoma), or incidental focal acantholytic dyskeratosis. The histopathologic analysis of the biopsies showed a focus of inframillimetric suprabasilar acantholysis with dyskeratosis in the epidermis. The acantholysis did not involve the full thickness of epidermis and spared the superficial layers of the epidermis and adnexal structures. Most importantly, there was no sign of breast carcinoma or radiation changes.

The histopathologic differential diagnosis essentially included an incidental focal acantholytic dyskeratosis (which can be seen in inflammatory and tumoral conditions),^7^ Grover disease (transient acantholytic dyskeratosis) in a Darier disease-like presentation, or Darier disease (keratosis follicularis). Focal acantholytic dyskeratosis and Grover disease usually have the same histopathologic presentation. Classically, there is acantholysis with suprabasilar clefts in the epidermis, associated with hyperkeratosis and/or parakeratosis, and a mixed dermal inflammatory cell infiltrate with or without dyskeratosis. Grover disease is unique among acantholytic disorders because it can histologically mimic any other acantholytic disorder by having a Darier disease-like presentation, a Hailey-Hailey disease-like presentation, a pemphigus vulgaris-like presentation, or a spongiotic-like presentation. Dyskeratosis with perinuclear halo (*corps ronds*) and pyknotic nuclei (*corps grains*) essentially defines Darier disease. It can sometimes be sporadic such as focal acantholytic dyskeratosis and Grover disease, but is more often an autosomal dominantly inherited disorder (mutations in the *ATP2A2* gene). The axillary location of the lesion could have been consistent with Hailey-Hailey disease (benign familial pemphigus), but not the presence of dyskeratosis and the extent of acantholysis limited to suprabasilar clefts instead of the full thickness of the epidermis. Clinical correlation is essential in making the correct diagnosis in this case because the histopathologic presentation of focal acantholytic dyskeratosis, Grover disease, and Darier disease could be identical. Although not available in our center, genetic analysis both on blood and skin samples would be useful for future studies to rule out a *forme fruste* of Darier or Hailey-Hailey disease, as well as a somatic postzygotic mutation.

The clinical differential diagnosis of a solitary lesion involving the axilla mainly includes Hailey-Hailey disease (benign familial pemphigus), granular parakeratosis, acanthosis nigricans, hidradenitis suppurativa, intertrigo, and tinea. Skin metastasis of breast cancer can present in many different forms including nodular metastasis, inflammatory metastasis (cellulitis-like), telangiectatic metastasis, “*peau d’orange”* metastasis (also referred as “like the skin of an orange” metastasis or as “*carcinoma en cuirasse*”), and alopecia neoplastica.

This case illustrates the importance of a good clinicopathologic correlation and highlights the fact that general pathologists may not recognize subtle details in skin biopsies.

## Conclusions

To the best of our knowledge, this is the first case report of an isolated and transient acantholytic and dyskeratotic lesion of the axilla. It could be a new variant of Grover disease and studies are needed to determine if it might be related to the ipsilateral breast carcinoma treatments. Future case reports would strengthen the possibility that this is a distinct entity.

This case is also a reminder of the significance of clinicopathologic correlation to determine the correct diagnostic and the proper management.

## Conflicts of interest

None disclosed.
